# Behaviour-specific habitat selection patterns of breeding barn owls

**DOI:** 10.1186/s40462-021-00258-6

**Published:** 2021-04-21

**Authors:** Robin Séchaud, Kim Schalcher, Ana Paula Machado, Bettina Almasi, Carolina Massa, Kamran Safi, Alexandre Roulin

**Affiliations:** 1 Department of Ecology and Evolution, University of Lausanne, Building Biophore, CH-1015 Lausanne, Switzerland; 2 Swiss Ornithological Institute, Seerose 1, 6204 Sempach, Switzerland; 3 Instituto de Investigación e Ingeniería Ambiental, Laboratorio de Ecología de Enfermedades Transmitidas por Vectores, Universidad Nacional de San Martín, 25 de Mayo, 1650 San Martín, Buenos Aires, Argentina; 4 Inmunova S.A., 25 de Mayo, 1650 San Martín, Buenos Aires, Argentina; 5 Department of Migration, Max Planck Institute of Animal Behaviour, Am Obstberg 1, 78315 Radolfzell, Germany; 6 Department of Biology, University of Konstanz, Universitätsstraße 10, 78464 Constance, Germany

**Keywords:** Agri-environment schemes, AES, Global positioning system technology, GPS, Home range, Path selection, Step selection, *Tyto alba*

## Abstract

**Background:**

The intensification of agricultural practices over the twentieth century led to a cascade of detrimental effects on ecosystems. In Europe, agri-environment schemes (AES) have since been adopted to counter the decrease in farmland biodiversity, with the promotion of extensive habitats such as wildflower strips and extensive meadows. Despite having beneficial effects documented for multiple taxa, their profitability for top farmland predators, like raptors, is still debated. Such species with high movement capabilities have large home ranges with fluctuation in habitat use depending on specific needs.

**Methods:**

Using GPS devices, we recorded positions for 134 barn owls (*Tyto alba*) breeding in Swiss farmland and distinguished three main behavioural modes with the Expectation-Maximization binary Clustering (EMbC) method: perching, hunting and commuting. We described barn owl habitat use at different levels during the breeding season by combining step and path selection functions. In particular, we examined the association between behavioural modes and habitat type, with special consideration for AES habitat structures.

**Results:**

Despite a preference for the most common habitats at the home range level, behaviour-specific analyses revealed more specific habitat use depending on the behavioural mode. During the day, owls roosted almost exclusively in buildings, while pastures, meadows and forest edges were preferred as nocturnal perching sites. For hunting, barn owls preferentially used AES habitat structures though without neglecting more intensively exploited areas. For commuting, open habitats were preferred over wooded areas.

**Conclusions:**

The behaviour-specific approach used here provides a comprehensive breakdown of barn owl habitat selection during the reproductive season and highlights its importance to understand complex animal habitat preferences. Our results highlight the importance of AES in restoring and maintaining functional trophic chains in farmland.

**Supplementary Information:**

The online version contains supplementary material available at 10.1186/s40462-021-00258-6.

## Introduction

The intensification of agricultural practices over the past century has severely changed European farmland [1, 2]. Many animal and plant populations declined [3, 4], and agriculture itself is suffering from the loss of services provided by wild organisms, such as crop pollination [5]. To counter the strong decrease in farmland biodiversity, European countries have adopted agri-environment schemes (AES), consisting mainly of paying direct subsidies to farmers to implement environmentally friendly farming practices. Despite documented beneficial effects on plant, insect and small mammal densities and on species richness [6–8], the effects of AES for larger vertebrate species remain unexplored. Probably the wide range of habitat used and movement capabilities render these species difficult to study. However, ensuring the presence of large vertebrate species, such as raptors, is an important step in the process of restoring and maintaining functional food chains in farmland ecosystems [9].

Distinguishing fine scale habitat preferences associated with different behaviours is key for understanding the underlying biological processes that drive animal movement [10]. How an animal uses its habitat is a complex decision-making process that can fluctuate with various factors, such as food availability [11], season [12], and individual life history traits [13], but also with the spatial and temporal scale considered [14, 15]. Habitat selection based on different behavioural modes has so far received limited attention, but the recent development in animal tracking technologies generated the opportunity to collect GPS locations at a high enough frequency to infer an animal’s behaviour from it [16–18]. Including a behavioural component in habitat selection analyses may be particularly valuable for depicting behaviour-specific selection patterns and consequently for improving prioritization in habitat management and conservation [10, 19].

The barn owl (*Tyto alba*), a raptor hunting small mammals in farmlands, suffered a rapid decline across Europe, mainly due to the spread of urbanization and the intensification of farming practices affecting the availability of nesting sites and quality of foraging habitats [20, 21]. While providing nest boxes relieved the shortage of secure breeding sites, the knowledge on habitat requirements is still patchy. A previous study did not find any association between nest box occupancy, reproductive success and the surrounding habitats [22], indicating that habitat preferences may occur at a finer scale within the home range. In a radio tracking study, Arlettaz et al. (2010) showed that foraging activity was more intense in cereal crops and grasslands than in more extensively exploited areas, suggesting that AES could be less important for farmland raptors than suspected.

Here, to explore the association between behavioural modes and habitat structure, we studied behaviour-specific habitat selection in wild barn owls breeding in an intensive agricultural landscape in western Switzerland. We expect barn owls to select different habitat structures according to the needs associated to the different behavioural modes. Over 2 years, we equipped barn owl breeding pairs with GPS devices. Combining the high location sampling rate with a behavioural segmentation method, we distinguished three main behavioural modes - perching, hunting and commuting – and related them with habitat use. These behavioural modes represent three main movement behaviours displayed by barn owls outside of their nests (their definition and manner distinction are further explained in the methods). To appropriately evaluate the use of rare and scattered habitats, as is the case with AES, we used step and path selection functions, which define the habitats available to an animal according to its current location. In this context, AES habitats might not be recognisably selected at the home range level but they can still be important components of barn owl behaviour-specific habitat use and possible key elements for farmland raptors.

## Materials and methods

### Study area and barn owl population monitoring

The study was carried out in 2016 and 2017 in a 1,000 km^2^ intensive agricultural landscape in western Switzerland where a wild population of barn owls breeds in nest boxes attached to barns [22]. In the first 2 weeks after hatching, the adult females remain almost entirely in the nest to provide warmth to their offspring and distribute the food among them brought by the male. After this period, both parents hunt small mammals, the male being the main contributor to the feeding of the nestlings [23].

### GPS tags and deployment

We used GiPSy-5 GPS tags (Technosmart, Italy) weighing approximately 12 g including battery (less than 5% of the owl body mass; in our population, body mass ranged from 251 to 393 g), measuring 30x20x10 mm including the battery, and coupled with a 40 mm long antenna. They were attached as backpacks with a Teflon harness. Each tag collected location and time every 10 s, from 30 min before dusk until 30 min after dawn, covering the entire owl nocturnal activity period.

Breeding barn owls were captured at their nest site when the oldest offspring was 25 days-old on average (SD = 2.8), equipped with GPS tags and released at the capture site. We recorded adults’ sex and age (categorized as yearlings or older birds). Approximately 2 weeks later, the owls were recaptured and the GPS tags recovered. The deployment of GPS tags corresponds to a period of intense habitat use by the parents to feed their nestlings, while being in accordance with ethical (earlier captures could lead to the abandonment of the clutch) and methodological constraints (later recapture could be compromised due to changes in food provisioning behaviour). Prior to any analysis, the 134 GPS tracks (72 males and 62 females) were filtered for aberrant positions using speed (excluding locations with a speed higher than 15 m/s) and location (excluding locations outside the study area). From the 1,924,623 collected positions, 1,922,636 were kept for the following analyses (1987 removed).

### Habitat monitoring

Once a barn owl breeding pair was equipped with GPS tags, we mapped the habitat at high-resolution in a radius of 1.5 km around the nest site and stored the surveys in QGIS v.2.18.13 [24]. The 7 km^2^ mapped corresponded to barn owl home range sizes reported in the literature (range: 0.9–8.1 km^2^) [21, 23, 25]. When barn owls travelled out of the area initially mapped, we went to the field to map it shortly after the GPS tag recovery. We adopted a 10-category habitat classification (Table S1), with 6 categories recorded directly in the field - cereals, root vegetables (sugar beets and potatoes), pastures, intensive meadows, extensive meadows and wildflower strips – and the four remaining categories – forests, forest edges, roads and settlements – were derived from the swissTLM^3D^ catalogue (Swiss Topographic Landscape Model). The cereals, root vegetables, intensive meadows and pastures habitat categories represent the intensive agricultural land use, whereas the wildflower strips and extensive meadows are AES implemented in the area to preserve and promote biodiversity in farmland (Table S2). Forests are common structural components of this landscape, and their edges are transitional zones between the forested areas and the crops. Finally, the farmland landscape is interspersed with anthropogenic constructions, which are represented here by the road and settlement habitat categories.

### Behaviour annotation

Barn owl movement data were classified into different behavioural modes using the Expectation-Maximization binary Clustering (EMbC) method implemented in the *EMbC* package [18]. EMbC is an unsupervised algorithm that clusters movement data based on speed and turning angle between locations. The three behavioural modes distinguished were perching, hunting and commuting. Perching, as a stationary behaviour, was characterized by low speed and a wide range of turning angles due to little GPS location errors. Hunting was characterized by low-medium speed and medium-high turning angles, whereas commuting was characterised by fast and straight flights. For validation, the EMbC behavioural classification was confronted to a visual classification performed on the tracks of 20 individuals. We found an average match of 92.7% between the visual and the EMbC classifications (perching: mean = 94.5%, SE = 2.3; hunting: mean = 92.6%, SE = 4.9; commuting: mean = 91.1%, SE = 3.8; San-Jose et al. 2019). These and all subsequent analyses were conducted with R v3.5.1 [26].

### Home range size and composition

Home range size was calculated using a 95% kernel density estimator method [27]. To deal with temporal auto-correlation between data points, we used the continuous-time movement modelling package (*ctmm*) [28] to calculate home range size via auto-correlated kernel density estimation (AKDE) [29]. The ctmm model was calibrated using User Equivalent Range Error (UERE), estimated with location data obtained by fixed GPS devices in open landscape. Model parameters with better fit were chosen automatically with the function *variogram.fit* in the *ctmm* package [28]. Barn owl home range composition was obtained by extracting the relative abundance of the 10 habitat categories contained in each home range.

We modelled the effect of sex and age, as well as year and date of GPS data collection, on the home range size of the barn owls using a linear mixed-effect model parametrized with the lmerTest package [30]. The home range size was log-transformed and the brood ID, grouping owls belonging to the same brood, set as random factor. For all linear mixed-effect models used in this paper, we checked for collinearity between predictors and verified the assumptions of Gaussian error distribution by visually inspecting residual diagnostic plots.

### Habitat selection

#### Home range selection

Home range selection (positioning of the home range in the landscape) compared the composition of the habitats available in the landscape, defined as the habitats contained in the 1.5 km radius around the nest site, with the ones contained in the home ranges (third-order selection; [31]), using *ADEhabitatHS* package [32]. For this and all subsequent analyses, selection ratios were estimated for each individual and habitat category, and then averaged to obtain the population’s habitat selection estimates. In addition, when some habitat selection coefficients were poorly estimated (because the habitats were absent or rare), we re-ran the model without the problematic habitat category to avoid misestimating the other selection estimates (Table S3).

#### Roosting and perching site selection

Roosting and perching site selection were analysed separately, with the former representing the sites used for hiding and resting during the day and the latter the sites used to perch during the night-time activity. Roosting site selection analyses compared the roosting sites’ habitats used to the ones available in the home range (third-order selection; [31]).

Perching site selection analyses compared night perching sites’ habitats to the ones available in the home range (third-order selection; [59]). Rather than considering each perching location point independently, they were grouped into perching events. As the choice of a perching site may depend on the surrounding landscape (perching sites corresponding to the nest site were excluded from the analyses), the habitat types present within a 100 m radius around the perching site were extracted.

#### Hunting ground selection

We parametrized a step selection function (SSF) to identify how habitat influences barn owl hunting movements [33]. The SSF considers the choice made by the animal at each step by comparing the observed step to a set of alternative ones, thus redefining the available habitats at every step. Using the *ctmm.fit* function in the *ctmm* package [28], we calculated that 30-s step time intervals were characterised by weak autocorrelation between steps, while maintaining sufficient resolution to address how habitat is selected during hunting. Once thinned to 30-s intervals, each observed step was paired with 100 alternative steps generated by randomly picking the step lengths (distance between successive locations) and turning angles (change in direction between steps) from the distribution of these parameters for the entire population using the *amt* package [34]. The habitat at the end point of each of these alternative steps was extracted. To compare habitat characteristics of observed and random steps, we ran a conditional logistic regression with *amt*’s *fit_clogit* function. The models contained 8 habitat categories – cereals, root vegetables, forests, forest edges, intensive meadows, extensive meadows, pastures and wildflower strips – as well as three movement parameters – step length, log of the step length and the cosine of the turning angle –known to render the estimates of the habitat regression parameters more robust [35, 36]. Including these movement variables as predictors allow to model both movement and habitat selection processes into an *integrated step selection function* [36]. The step and burst IDs were entered as strata in the model, the first one to link the real and the 100 alternative steps and the second one to group the steps belonging to the same track. Two habitat categories – roads and settlements – were not included in the models because they were too rare in the dataset and prevented the models from converging.

To investigate if the similarity in habitat selection coefficients between individuals was related to seasonality and individual factors, we used a non-metric multi-dimensional scaling approach (NMDS; [37]). NMDS is a rank-based ordination method that uses pairwise distances between objects or individuals, and represents them in a low dimensional space. Using the coefficients of selection, a dissimilarity matrix was built with the Bray-Curtis method using the function *metaMDS* in the *vegan* package [38]. The wildflower strips habitat category was removed from this analysis because the limited number of coefficients obtained was not sufficient to parameterize a valid NMDS model (Table S3). To investigate if the year and date of GPS data collection, as well as the sex and the age of the owl, could explain the similarity or differences between birds in the classification proposed by the NMDS, we ran a permutation test using the *envfit* function in the same package with 10,000 permutations. We included in the model the year and date of GPS data collection as proxies for temporal variations in the landscape structure and profitability.

#### Commuting path analyses

Commuting tracks were classified in three main categories, each with a different purpose. The first type of commuting flight takes place when an owl leaves its nest box to reach a hunting ground or a perching spot. The second one is the reverse, when the owl catches a prey item and returns to the nest box to feed its nestlings. The third type of commuting is used to move within the landscape, to travel between hunting or perching sites, and is independent from the nest box location.

We built a path selection function (PathSF) to investigate the influence of landscape on commuting flights [39]. In PathSF, the entire path is the unit of measurement and, in a similar fashion to SSF, is compared to randomly generated paths. We discarded the commuting to and from the nest box to avoid the bias associated to the habitats surrounding the nest box location, and therefore considered only the commuting flights within the habitat. For each observed path, 20 alternative paths were generated by first randomly relocating the starting point of the path within a radius of 1.5 km, and then by rotating it by a random angle between 0 and 360 degrees [39]. Habitats contained in a 20-m buffer along the tracks were extracted and, to compare observed and random paths, conditional logistic regressions were built using the *fit_clogit* function in the *amt* package [34]. For statistical purposes, we grouped cereals, vegetable roots, pastures and intensive meadows into an “intensive open habitats” category, while the categories extensive meadows and wildflower strips were aggregated into “extensive open habitats”. We built conditional logistic regression models containing intensive open habitats, extensive open habitats, forests, forest edges and roads as explanatory variables, and the burst ID as strata. Settlements were excluded from the analyses because they were under-represented in the extracted habitats and caused models to not converge.

For each of the three commuting flight types, the deviation from the straightest path was measured as the difference between the length of the real track and that of the shortest path between the starting and ending point of the commuting event. To test if the distance covered, the deviation from the straightest path, and the speed (calculated as the distance covered divided by the time) differed between the three types of commuting, we ran linear mixed-effect models using the *lmerTest* package [30]. The distance covered and the deviation from the straightest path were log-transformed. The type of commuting was entered as an explanatory variable and the bird identity set as random factor.

## Results

### Behaviour characteristics and activity period

For the 134 individuals considered (72 males and 62 females), the number of nights with data recorded varied between 4 and 15 (mean = 9.9; SD = 2.1) and the time interval between each location was 9.9 s on average (SD = 1.3). Behavioural annotation of the GPS tracks (sampling every 10 s) revealed great differences in step lengths and turning angles between perching, hunting and commuting behavioural modes (Fig. S1). Hunting flights were performed at an average speed of 4.9 m/s (SD = 1.0; range: 1.7–12.2 m/s), while the mean speed of commuting flights was 6.6 m/s (SD = 1.1; range: 2.4–13.4 m/s). Occasionally, owls flying at speeds above 10 m/s were recorded when commuting (max = 13.4 m/s), for a flight duration from 50 s to 9 min (Fig. S2).

The nightly activity period, defined as the time between two daylight roosting events, varied from 5.4 min to 10.4 h (median = 6.8 h, SD = 2.1; Fig. S3). During their activity period, barn owls perched on average 77.5% (SD = 13.8; range: 14.6–100%) of the time, while the rest was composed of 12.7% of hunting (SD = 9.4; range: 0–75.2%) and 9.8% of commuting (SD = 7.4; range: 0–53.3%; Fig. S4).

### Home range size and composition

Home range size varied significantly (mean = 6.6 km^2^; range: 0.96–25.46; Fig. S5), with males having smaller home ranges than females. On the other hand, neither age, year nor date were related to the home range size (Table 1).

**Table 1:** Effect of individual and time parameters on barn owl home range size. Results from a linear-mixed model on 134 log-transformed home range sizes from 83 broods (set as random factor)

Parameter	Estimate ± SE	df	t-value	***p***
Intercept	2.028 ± 0.120	120.76	16.94	< 0.001
Sex^a^	−0.440 ± 0.102	68.77	−4.32	< 0.001
Date^b^	−0.032 ± 0.061	86.55	− 0.53	0.598
Year^a^	−0.166 ± 0.122	75.38	−1.37	0.176
Age^a^	−0.135 ± 0.117	128.90	−1.16	0.249

^a^ Males minus females; 2017 minus 2016; older birds minus yearlings

^b^ The Date parameter was scaled

**Fig. 1:**
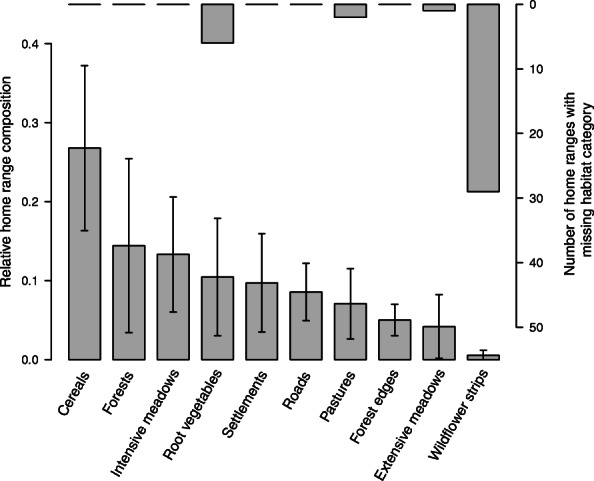
Habitat composition of barn owl home ranges. For each of the 10 habitat categories, population mean and associated standard deviations are shown on the left axis, and the number of home ranges with missing habitat category on the right axis (134 home ranges in total). The habitats are ordered from the most to the least abundant

Despite large inter-individual variations (Fig. 1), barn owl home ranges contained consistently and predominantly intensive agricultural fields (24.6% of cereals, 11.5% of intensive meadows, 10.4% of root vegetables and 7.1% of pastures). The forested areas were the second most represented habitat class (18.1% of forest and 5% of forest edges), followed by human-made constructions (10.9% of settlements and 8.4% of roads). Finally, extensively exploited areas were the rarest habitat class, with 4.1% of extensive meadows and 0.5% of wildflower strips. In addition to being the rarest habitat, wildflower strips were also absent in 21% of the home ranges (Fig. 1).

### Home range selection

**Fig. 2:**
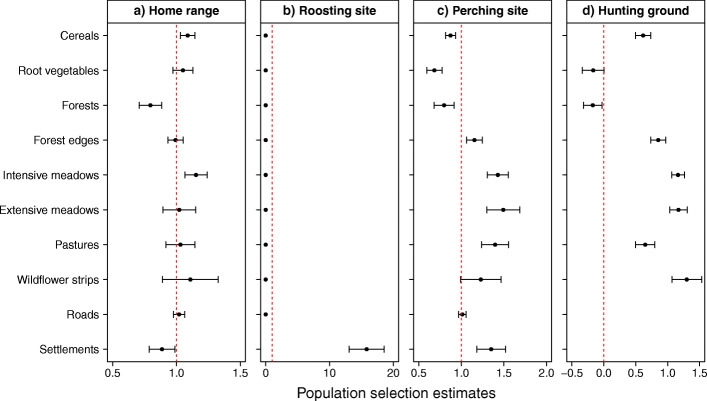
Habitat selection population estimates. Home range composition, roosting and perching site selection analyses were computed following the Manly’s third-order selection approach. Hunting ground selection followed the step-selection function (SSF) approach. Models were run for every individual and then averaged to obtain population estimates (mean and associated 95% confidence intervals are shown). Estimates on the right and left side of the dotted red line indicate, respectively, selected and avoided habitats

At the home range level, habitat selection revealed that barn owls incorporated in their home range some habitat types in disproportion compared to surrounding landscape (Fig. 2a). Intensive meadows and cereals were found in higher proportion in home ranges than in the nearby landscape, whereas settlements and forests were included in the home ranges in a smaller proportion than available. The selection ratios for the other habitat categories did not differ significantly from random use.

### Roosting and perching site selection

Over the 915 daylight roosting events identified, 909 were located in barns or farms (468 in the nest box or in the nest box building, 441 in another building) and 6 were in forested areas, resulting in a clear selection pattern for settlements and avoidance of all 9 other habitat types (Fig. 2b). Roosting in natural habitats is thus an extremely rare event, concerning here 3 different females (out of 134 birds).

Overall habitat selection for night-time perching showed a clear pattern of habitat selection and avoidance (Fig. 2c). Among the habitats selected for perching, extensive meadows had the highest selection ratio, followed by intensive meadows, pastures, settlements and forest edges, while cereals, root vegetables and forests were avoided. Finally, roads and wildflower strips’ selection ratios indicated a use according to their availability.

### Hunting ground selection

The hunting SSF model revealed clear differences in selection ratios between the different habitat categories (Fig. 2d). Hunting owls avoided forests, and the root vegetables to a lesser extent, while selecting all six remaining habitat categories. Among the selected habitats, wildflower strips, extensive and intensive meadows were the most preferred ones, followed by forest edges, pastures and cereals. The scaled averaged estimates of the three movement parameters included in the models to increase the robustness of the habitat estimates were 0.04 for the cosine of the turning angle (SD = 0.15), − 0.10 for the step length (SD = 0.39) and 0.21 for the log of the step length (SD = 0.53).

**Fig. 3:**
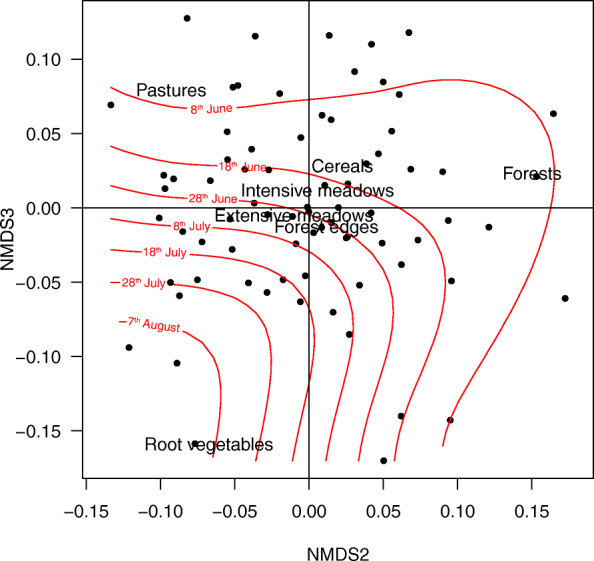
Non-metric multi-dimensional scaling (NMDS) plot of hunting habitat selection estimates, each dot representing an individual. The effect of the date (in red) in the dimensions 2 and 3 (encompassing most of the date influence) is shown. Habitat categories are plotted for ease of understanding

The three-dimensional NMDS model was associated with a stress value of 0.15, indicating a reliable representation of the coefficients of selection (Fig. S6). The first NMDS dimension contrasted between intensive meadows to root vegetables and forests, the second distinguished the forests, and the third one the root vegetables (Table S4). We tested if the similarity in habitat selection between individuals was related to the year and date of GPS installation, two proxies for structural and qualitative modifications of the landscape, and the owl sex and age, two parameters associated with individual investment and hunting experience. The permutation test showed a significant effect of the date (*r*^*2*^ = 0.24, *p* <  0.001), whereas the year (*r*^*2*^ = 0.02, *p* = 0.19), sex (*r*^*2*^ = 0.03, *p* = 0.12) and age (*r*^*2*^ = 0.03, *p* = 0.11) showed no significant relationship with hunting coefficient variations. The dimensions 2 and 3 of the NDMS encompassed most of the effect of the date (NMDS1 = -0.16, NMDS2 = 0.59, NMDS3 = -0.79), indicating higher root vegetable selection ratios at the end than at the beginning of the season, whereas the opposite was observed for forests (Fig. 3).

### Commuting path analyses

**Fig. 4:**
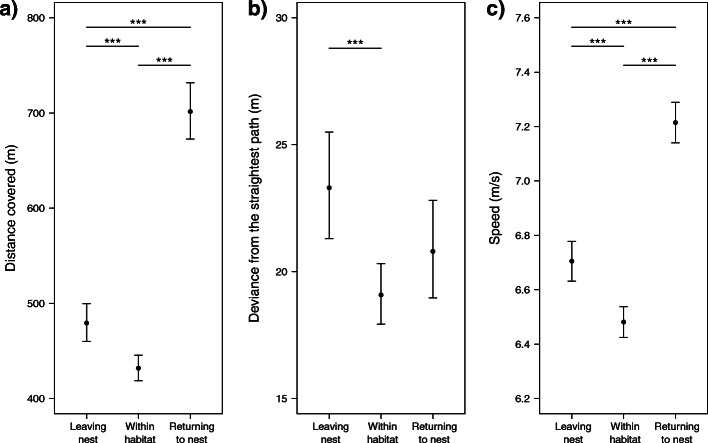
Comparison of three type of commuting flights (leaving the nest, commuting within the habitat, and returning to the nest). Panel **a**) shows the distance covered, **b**) the deviance from the straightest path, and **c**) the flight speed. For each flight type, the mean and 95% confidence intervals are shown

The within habitat commuting PathSF model showed that all habitats considered were used for commuting, except for forest which was clearly avoided (Table 2). Considering all commuting flight types, owls covered a median distance of 447.7 m (range: 97.9–3676.1). The longest commuting flights were performed when returning to the nest, followed by the flights to leave it and the smallest distances covered were within the habitat (Fig. 4a, Table 3). When commuting, owls deviated 20.5 m (range: 0.1–991.2) on average from the most direct path. They deviated more from the straightest path when leaving the nest box than when they commuted in the habitat (Fig. 4b, Table 3). When commuting, owls flew at an average speed of 6.5 m/s (range: 3.4–13.4). They commuted the fastest to return to the nest box, followed by leaving it, and lastly within the habitat (Fig. 4c, Table 3).

**Table 2:** Commuting path selection. Using the path selection function approach (PathSF), selection ratios for each individual and habitat were extracted from a conditional logistic regression model including the five habitat categories listed and the burst as strata. Mean population selection estimates and associated 95% CI are shown, and the habitats are ordered from the most to the least preferred

Habitat	Selection ratio	Lower CI	Upper CI
Open intensive habitats	2.10	1.70	2.50
Roads	1.69	0.87	2.51
Open extensive habitats	1.52	1.04	2.01
Forest edges	1.05	0.24	1.85
Forests	−0.61	−1.19	−0.03

**Table 3:** Difference between the three types of commuting – leaving (L) the nestbox, returning (R) to it and within (W) the habitat – in the distance covered, deviance from the straightest path and flight speed. Results from linear-mixed models including 12,503 tracks from 134 barn owls (owl identity set as random factor). The distance covered and the deviance from the straightest path were log-transformed

Parameter	Estimate ± SE	df	t-value	***p***
**Distance covered**				
L - W	−0.104 ± 0.017	12,470	−6.20	< 0.001
L - R	−0.381 ± 0.022	12,412	17.30	< 0.001
W - R	−0.485 ± 0.0174	12,461	27.93	< 0.001
**Deviance from the straightest path**				
L - W	−0.199 ± 0.039	12,490	−5.02	< 0.001
L - R	−0.114 ± 0.052	12,439	−2.18	0.078
W - R	−0.086 ± 0.041	12,484	2.09	0.101
**Speed**				
L - W	−0.224 ± 0.028	12,468	−7.83	< 0.001
L - R	−0.510 ± 0.037	12,411	13.62	< 0.001
W - R	−0.733 ± 0.029	12,454	24.83	< 0.001

## Discussion

In the context of preserving biodiversity in farmlands, our study provides a comprehensive breakdown of barn owl habitat selection during the reproductive season. The various behaviour-specific habitat analyses highlight the complementarity of this approach in understanding complex animal habitat preference and for proposing targeted conservation actions.

With an average size of 6.6 km^2^, the home range sizes obtained in our study correspond to the ones previously described for barn owls in Europe [21, 25, 40, 41]. In this species, parental investment varies between sexes [42, 43], which is consistent with our finding that males had smaller home ranges than females. The bigger home range of females could be explained by double-brooded females which often desert their first brood to start a new one elsewhere with another mate [42, 44]. To find a new partner, females may prospect large areas, while their first male is still hunting close to their first nest.

Forested areas, commonly known to be avoided by barn owls, were under-represented in barn owl’s home ranges, probably because its morphology (i.e. short tail and long wings) and hunting-on-the wing technique limits its use of closed habitats [21, 23]. Thus, home ranges contained mainly open habitats, with the most common ones - cereals and intensive meadows - being preferentially included (Fig. 2a). AES habitat categories were not selected at the home range level. Increasing the proportion of AES, the least represented habitat with low connectivity between each patch, in the home range likely implies the inclusion of the more abundant habitat categories.

Despite selection at the home range level being characterized by a preference for the most common habitats, behaviour-specific analyses revealed distinctive habitat use depending on the behavioural mode. During the day, barn owls roosted almost exclusively in buildings despite the apparent availability of natural sites (Fig. 2b). They might use the urban environment to shelter against adverse weather conditions, minimize the energy invested to thermoregulate and reduce the risk of predation or disturbance by competitors [45, 46].

During the night, barn owls preferred to perch in meadows, pastures, settlements and along forest edges (Fig. 2c). Perching habitat selection pattern was fairly similar to that of hunting, hinting at the use of the sit-and-wait hunting technique seen in many raptors [21, 47]. It may also reflect an opportunistic behaviour, in which resting or preening close to hunting grounds could offer the opportunity to capture a prey [48, 49]. In addition to the natural perching sites, barn owls also benefit from the fencing of pastures and artificial poles that are installed by farmers to attract raptors as pest-control agent [50].

For hunting, barn owls displayed a strikingly contrasted selection pattern, with habitats being either preferred or avoided but not neutral (i.e. used at the same frequency as availability; Fig. 2d). Surprisingly, most habitats were actually selected as hunting grounds, with a wide range of vegetation structure, prey abundance and agricultural regimes, reflecting the species’ flexibility and adaptability. In a previous study, Arlettaz et al. (2010) showed a preference for cereals and intensive meadows (referred to as grassland in their study), arguing that vegetation structure was more important than prey availability. Our results confirm a selection for these habitats as hunting grounds, but also highlight the importance of extensive meadows and wildflower strips, the rarest but most preferred hunting habitats. The habitats selected for hunting differ strongly in vegetation height, and we found no seasonal selection differences in habitats with large fluctuations in vegetation structure throughout the year. Therefore, we could not find a limitation of habitat use based on vegetation structure as previously proposed [7, 51]. Further research should investigate the interconnected effects of vegetation structure and prey density on hunting ground selection and success, while accounting for individual specific foraging strategies (on the wing or perched). In addition, as barn owls display a plumage colour polymorphism [23], upcoming studies should investigate morph-specific habitat preferences and foraging strategies, specifically in relation to night illumination [52].

Similarly to the other behavioural modes, commuting tracks bypassed the forested areas (Table 2). Flying over such tall structures as forest would possibly require a larger energetic investment for this usually low-flying bird [21]. Commuting tracks followed nearly straight paths and are hence optimised to reach their destination at high speed as directly as possible (Fig. 4). Since the flights to leave the nest box are shorter than those to return to it, owls might gradually move away from their nest box during the hunt. As central place foragers carrying one prey per nest visit, it would be advantageous for the owls to optimize their energy expenditure by starting to hunt close to the nest [53, 54]. Although most commuting flights were almost straight, some specific tracks deviated considerably from the shortest route (up to 991 m of difference), possibly due to fine-scale environmental or habitat structure variations. Avoiding adverse conditions such as strong head-winds or taking advantage of potential uplifts along tall structures could justify taking a longer route while optimizing energy expenditure [55].

## Conclusions

This study highlights the need of behaviour-specific analyses to understand complex animal habitat preferences. The combination of the results unveils the barn owl as a generalist and opportunistic bird, with plastic behaviour to exploit a variety of open habitats in a farmland landscape. In comparison with a previous study [51], our results showed that barn owls select AES habitats, such as wildflower strips and extensive meadows, as hunting grounds. This supports the importance of such schemes to restore and maintain functional trophic chains in farmland, and stresses the need to promote such measures that are still rare and scattered. The quality of these areas dedicated to biodiversity could also be improved by increasing the connectivity between these plots [56, 57]. In addition, their use by raptors could be enhanced through the installation of artificial poles in dense vegetation to favour the use of the sit-and-wait hunting technique [58, 59]. Future analyses should investigate the profitability of AES for farmland raptors, by translating AES availability and use into fitness benefits. Finally, our work demonstrates the importance of addressing habitat selection on a behaviour-specific perspective to account for the complex animal habitat selection patterns when proposing appropriate conservation plans.

## Supplementary Information

**Additional file 1: Table S1.** For each habitat category are given the source of the data, and the object with the associated buffer used for creating the layers. **Table S2.** Correspondence between habitat classification and official agri-environment schemes (AES) categories. **Table S3.** Number of barn owl individuals included in habitat selection models. **Table S4**. Correspondence between habitat categories and the three dimensions of the non-metric multi-dimensional scaling (NMDS) performed on hunting selection estimates. **Fig. S1.** Step length and turning angle distributions for the perching, hunting and commuting behaviours. **Fig. S2.** Relation between hunting and commuting flight speeds and the behavioural event duration. **Fig. S3.** Distribution of night activity period duration, defined as the time between two daylight roosting events. **Fig. S4.** Proportion of activity time per night spent perching, hunting or commuting. **Fig. S5.** Home range size in relation to barn owl sex. **Fig. S6.** Non-metric multi-dimensional scaling (NMDS) model parametrization.

## Data Availability

The GPS datasets generated and analysed during the current study are available in Movebank (www.movebank.org), under the project named “Barn owl (*Tyto alba*)” (ID 231741797). The habitats maps produced during the current study are available from the corresponding author on reasonable request.
