# Supplementary material for: Behaviour-specific habitat selection patterns of breeding barn owls
Source: Mov Ecol. 2021 Apr 21;9:18. doi: 10.1186/s40462-021-00258-6 (PMC8059222; doi:10.1186/s40462-021-00258-6)
Supplement: Supplementary file 1 — Additional file 1: Table S1. For each habitat category are given the source of the data, and the object with the associated buffer used for creating the layers. Table S2. Correspondence between habitat classification and official agri-environment schemes (AES) categories. Table S3. Number of barn owl individuals included in habitat selection models. Table S4. Correspondence between habitat categories and the three dimensions of the non-metric multi-dimensional scaling (NMDS) performed on hunting selection estimates. Fig. S1. Step length and turning angle distributions for the perching, hunting and commuting behaviours. Fig. S2. Relation between hunting and commuting flight speeds and the behavioural event duration. Fig. S3. Distribution of night activity period duration, defined as the time between two daylight roosting events. Fig. S4. Proportion of activity time per night spent perching, hunting or commuting. Fig. S5. Home range size in relation to barn owl sex. Fig. S6. Non-metric multi-dimensional scaling (NMDS) model parametrization. [file 40462_2021_258_MOESM1_ESM.docx]

**Table S1.** For each habitat category are given the source of the data, and the object with the associated buffer used for creating the layers.

| **Habitat category** | **Source** | **Object** | | **Buffer** | |  |
| --- | --- | --- | --- | --- | --- | --- |
| Cereals | Field monitoring | - | | - | |  |
| Root vegetables | Field monitoring | - | | - | |  |
| Pastures | Field monitoring | - | | - | |  |
| Intensive meadows | Field monitoring | - | | - | |  |
| Extensive meadows | Field monitoring | - | | - | |  |
| Wildflower strips | Field monitoring | - | | - | |  |
| Forests | SwissTLM^3D^ | TLM_BODENBEDECKUNG | |  | |  |
|  |  |  | OBJEKTART = Wald | | - | |
|  |  |  | OBJEKTART = Wald offen | | - | |
|  |  |  | OBJEKTART = Gebueschwald | | - | |
| Forest edges | SwissTLM^3D^ | Buffer around "Forests" layer | | 10 m | |  |
| Roads | SwissTLM^3D^ | TLM_STRASSE | |  | |  |
|  |  |  | OBJEKTART = Autobahn | | 25 m | |
|  |  |  | OBJEKTART = Autostrasse | | 15 m | |
|  |  |  | OBJEKTART = 10m Strasse | | 10 m | |
|  |  |  | OBJEKTART = 8m Strasse | | 8 m | |
|  |  |  | OBJEKTART = 6m Strasse | | 6 m | |
|  |  |  | OBJEKTART = 4m Strasse | | 4 m | |
|  |  |  | OBJEKTART = 3m Strasse | | 3 m | |
|  |  |  | OBJEKTART = 2m Weg | | 2 m | |
|  |  |  | OBJEKTART = 1m Weg | | 1 m | |
| Settlements | SwissTLM^3D^ | TLM_GEBAEUDE_FOOTPRINT | |  | |  |
|  |  |  | OBJEKTART = all | | 20 m | |

**Table S2.** Correspondence between habitat classification and official agri-environment schemes (AES) categories. The official AES census performed by the canton Vaud in 2017 was compared to 24 corresponding vegetation maps mapped in this study (selected based on their location and year of mapping). The AES category identifiers correspond to the ones defined by the Federal Office for Agriculture (FOAG): 556 = Floral fallow; 557 = Rotational fallow; 559 = Extensive herbaceous strips; 611 = Extensive meadows; 612 = Low intensity meadows; 617 = Extensive pastures; 852 = Hedges and copses. Percentage of correspondence are indicated with the number of matching parcels in brackets.

|  | **AES category identifiers** | | | | | | | **non-AES** | **Total** |
| --- | --- | --- | --- | --- | --- | --- | --- | --- | --- |
|  | **556** | **557** | **559** | **611** | **612** | **617** | **852** |  |  |
| **Wildflower strips** | 70.23% (92) | 26.72% (35) | 2.29% (3) | 0.76% (1) | 0 | 0 | 0 | 0 | 131 |
| **Extensive meadows** | 0.17% (1) | 0.50% (3) | 0.17% (1) | 93.68% (563) | 1.83% (11) | 1.83% (11) | 1.32% (8) | 0.50% (3) | 601 |
| **Intensive meadows** | 0 | 0 | 0 | 5.44% (102) | 5.39% (101) | 0.96% (18) | 0 | 88.21% (1653) | 1874 |
| **Pastures** | 0 | 0 | 0 | 8.96% (82) | 0 | 18.80% (172) | 1.09% (10) | 71.15% (651) | 915 |

**Table S3.** Number of barn owl individuals included in habitat selection models. For each analysis, poorly estimated coefficients (because the habitat category was absent or too rare) were removed from the models to avoid misestimating the other habitat selection estimates. In total, 134 barn owls were included in the study.

| **Category** | **Home range** | **Roosting site** | **Perching site** | **Hunting ground** |
| --- | --- | --- | --- | --- |
| Cereals | 134 | 134 | 134 | 134 |
| Root vegetables | 122 | 128 | 127 | 114 |
| Forests | 134 | 134 | 134 | 117 |
| Forest edges | 134 | 134 | 134 | 131 |
| Intensive meadows | 134 | 134 | 134 | 132 |
| Extensive meadows | 131 | 133 | 132 | 115 |
| Pastures | 132 | 132 | 132 | 118 |
| Wildflower strips | 115 | 104 | 104 | 62 |
| Roads | 134 | 134 | 134 | - |
| Settlements | 134 | 134 | 134 | - |

**Table S4**. Correspondence between habitat categories and the three dimensions of the non-metric multi-dimensional scaling (NMDS) performed on hunting selection estimates.

| **Habitat** | **NMDS 1** | **NMDS 2** | **NMDS 3** |
| --- | --- | --- | --- |
| Cereals | -0.033 | -0.023 | -0.028 |
| Root vegetables | 0.146 | 0.070 | 0.160 |
| Forests | 0.138 | -0.150 | -0.021 |
| Forest edges | -0.062 | -0.011 | 0.014 |
| Intensive meadows | -0.114 | -0.006 | -0.012 |
| Extensive meadows | -0.005 | 0.010 | 0.004 |
| Pastures | 0.092 | 0.092 | -0.081 |

**Fig. S1.** Step length and turning angle distributions for the perching, hunting and commuting behaviours. The step length is in meters and the turning angle in radians, with a time interval between each location of 10 seconds.


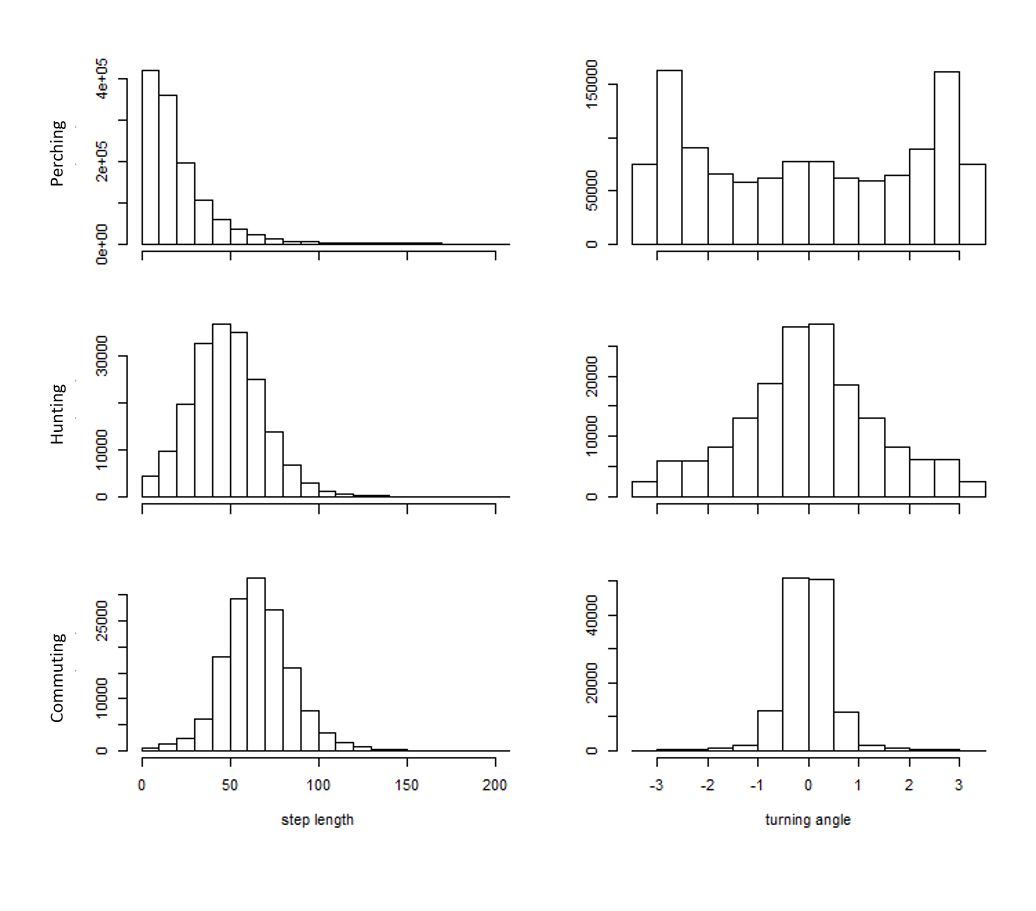


**Fig. S2.** Relation between a) hunting and b) commuting flight speeds and the behavioural event duration.

**
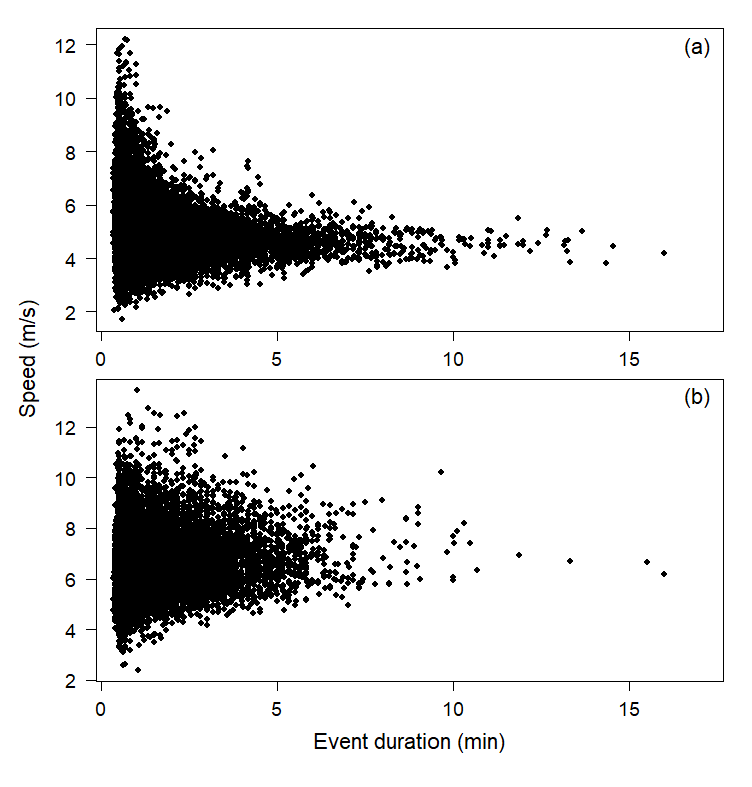
**

**Fig. S3.** Distribution of night activity period duration, defined as the time between two daylight roosting events.

**
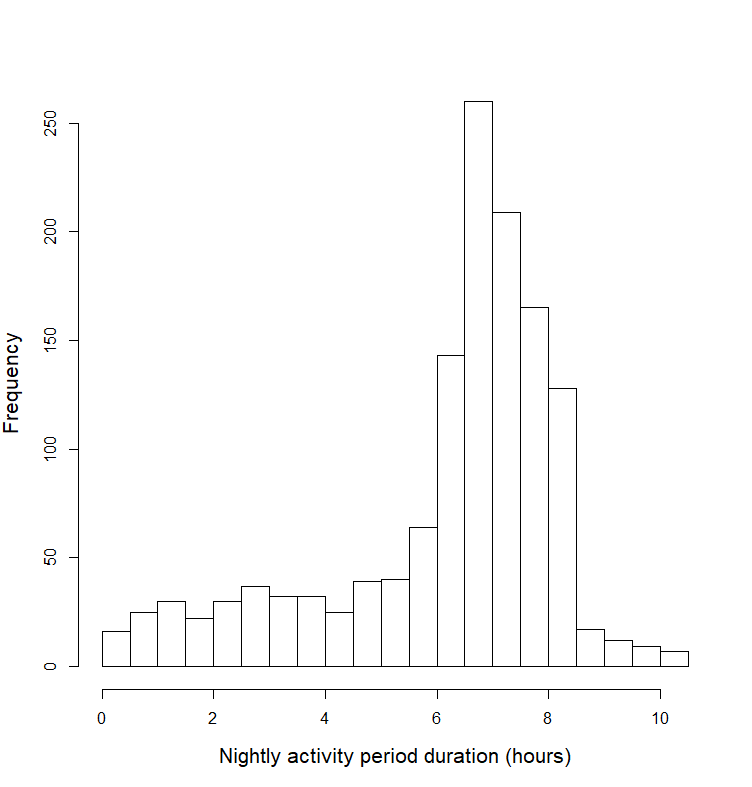
**

**Fig. S4.** Proportion of activity time per night spent perching, hunting or commuting.


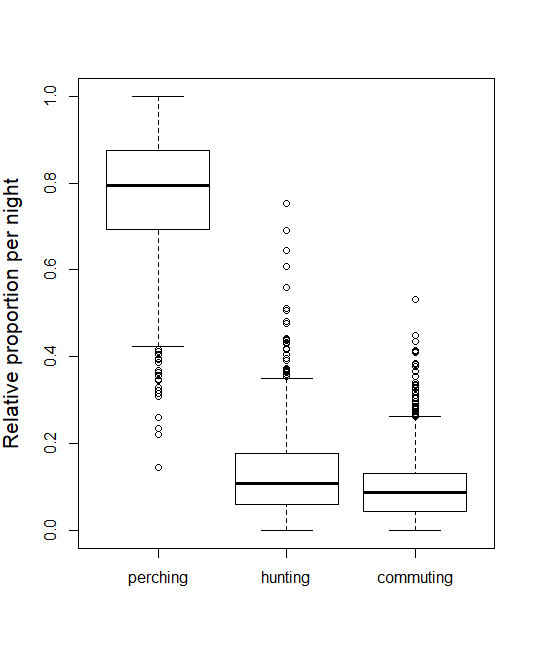


**Fig. S5.** Home range size in relation to barn owl sex.

**
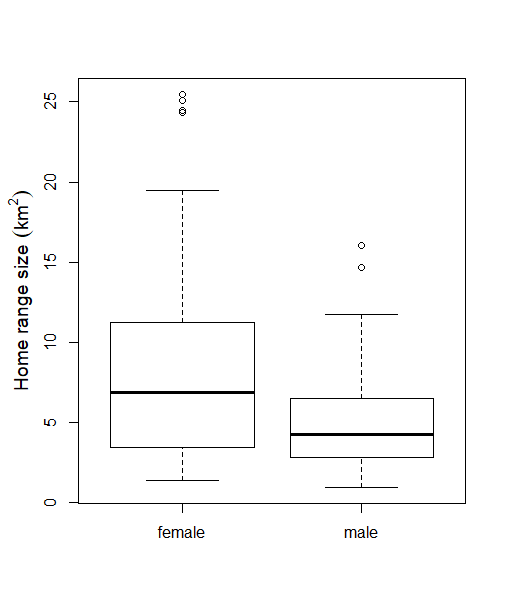
**

**Fig. S6.** Non-metric multi-dimensional scaling (NMDS) model parametrization. NMDS was built in three dimensions, resulting in a stress value of 0.15 and an acceptable fit.

**
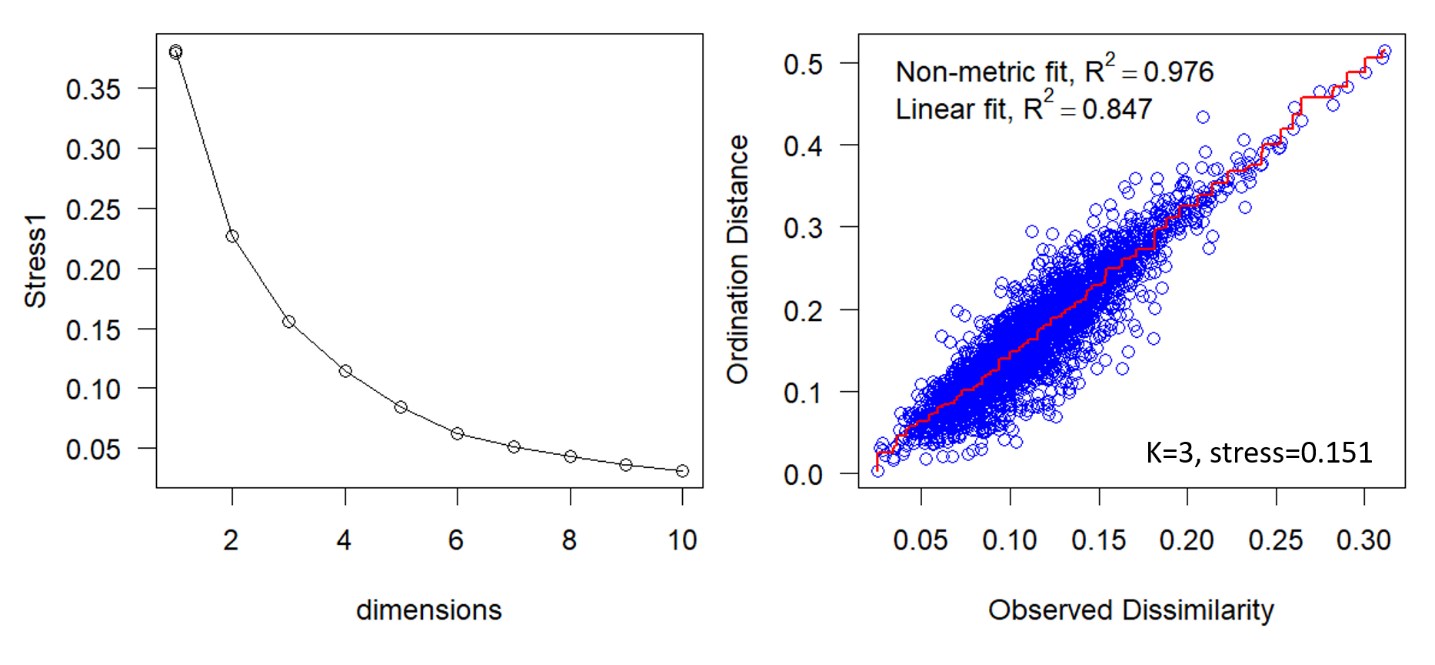
**
